# Supplementary material for: Influence of aluminium distribution on the diffusion mechanisms and pairing of [Cu(NH3)2]+ complexes in Cu-CHA
Source: Nat Commun. 2025 Jan 11;16:603. doi: 10.1038/s41467-025-55859-1 (PMC11724864; doi:10.1038/s41467-025-55859-1)
Supplement: Supplementary file 1 — Supplementary information [file 41467_2025_55859_MOESM1_ESM.pdf]

# Supplementary Information: Influence of aluminium distribution on the diffusion mechanisms and pairing of $[Cu(NH_3)_2]^+$ complexes in Cu-CHA

Joachim D. Bjerregaard[1,2,*], Martin Votsmeier[2], and Henrik Grönbeck[1,*]

[1]Department of Physics and Competence Centre for Catalysis, Chalmers University of Technology, SE-412 96 Göteborg, Sweden

[2]Umicore AG & Co. KG, Rodenbacher Chaussee 4, 63457 Hanau, Germany

[*]Corresponding authors: joabje@chalmers.se;ghj@chalmers.se

# Supplementary Methods

## Training data

The structures used for training the machine learning force field (ML-FF) cover a broad range of different Si/Al, Cu/Al ratios, sizes of supercells, and Al distributions. This is important as the objective of the ML-FF is to be able to predict forces and energies, for large supercells with different Si/Al, Cu/Al ratios for which the force field has not been trained. The training process follows an iterative approach illustrated in Figure 1. Firstly, a preliminary force field is trained using structures from *ab initio* MD. Secondly, ML-FF MD simulations at up to 300 °C are performed to generate new structures, which are evaluated with single-point DFT calculations. The ML-FF MD simulations are augmented with metadynamics simulations of $[Cu(NH_3)_2]^+$ diffusion through the eight-membered ring in CHA to ensure adequate sampling of crucial events. 200 and 500 structures are typically uniformly extracted from the ML-FF MD trajectories for the single point calculations. 90 % of the structures are used for training, whereas the 10 % are used for validation.

Throughout the iterative process, the complexity of the structures is increased, incorporating a higher number of Al, $NH_4^+$ and $[Cu(NH_3)_2]^+$ ions as the force field becomes more stable. The final data set for training consists of 52481 single-point calculations on 160 different systems. A list of training data is reported in Table 1, which spans from a Si/Al ratio of 107 to 4.4. Note that the Al ions are randomly distributed and the row *Al distributions* indicate how many different distributions that are used.

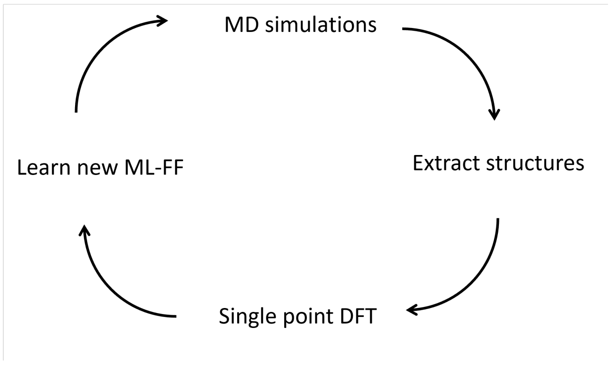


Supplementary Fig. 1: Illustration for the iterative procedure when training the machine learning force field.

Supplementary Table 1: Overview of systems included in the training data of the ML-FF. The supercells are constructed from the hexagonal unit cell of CHA having 36 Si+Al atoms and 72 O atoms.

| Supercell | Si/Al | Cu/Al | $[Cu(NH_3)_2]^+$ | $NH_4{}^+$ | Al distributions | Single point |
|---|---|---|---|---|---|---|
| 1×1×1 | - | - | 0 | 0 | - | 450 |
| 1×1×1 | 35 | 1 | 1 | 0 | 1 | 1443 |
| 1×1×1 | 35 | 0 | 0 | 1 | 1 | 1141 |
| 1×1×1 | 17 | 1 | 2 | 0 | 3 | 1303 |
| 1×1×1 | 11 | 1 | 3 | 0 | 1 | 180 |
| 1×1×1 | 17 | 0 | 0 | 2 | 2 | 702 |
| 1×1×1 | 8 | 0 | 0 | 4 | 1 | 261 |
| 1×1×1 | 6.2 | 0 | 0 | 5 | 1 | 261 |
| 1×1×1 | 17 | 0.5 | 1 | 1 | 2 | 1053 |
| 1×1×1 | 11 | 0.67 | 2 | 1 | 1 | 351 |
| 1×1×1 | 8 | 0.5 | 2 | 2 | 1 | 670 |
| 1×1×1 | 11 | 0.33 | 1 | 2 | 1 | 180 |
| 2×1×1 | 71 | 1 | 1 | 0 | 3 | 4231 |
| 2×1×1 | 71 | 0 | 0 | 1 | 2 | 3150 |
| 2×1×1 | 35 | 1 | 2 | 0 | 6 | 4818 |
| 2×1×1 | 35 | 0 | 0 | 2 | 5 | 6046 |
| 2×1×1 | 35 | 0.5 | 1 | 1 | 3 | 3071 |
| 2×1×1 | 23 | 0.33 | 1 | 2 | 4 | 5321 |
| 2×1×1 | 23 | 0.67 | 2 | 1 | 3 | 3593 |
| 2×1×1 | 23 | 1 | 3 | 0 | 3 | 2940 |
| 2×1×1 | 11 | 0.67 | 4 | 2 | 4 | 1794 |
| 2×1×1 | 6.2 | 0.5 | 5 | 5 | 4 | 1798 |
| 3×1×1 | 107 | 1 | 1 | 0 | 1 | 1074 |
| 3×1×1 | 107 | 0 | 0 | 1 | 1 | 440 |
| 3×1×1 | 8 | 0.5 | 6 | 6 | 3 | 1350 |
| 3×1×1 | 6.7 | 0.71 | 10 | 4 | 3 | 1350 |
| 3×1×1 | 5.75 | 0.5 | 8 | 8 | 3 | 1350 |
| 3×1×1 | 5.75 | 0.13 | 2 | 14 | 3 | 1260 |
| 3×1×1 | 4.4 | 0.3 | 6 | 14 | 2 | 900 |

## Validation of the force field

The typical method for validating a machine learning force field (ML-FF) involves comparing ML-FF predicted energies and forces with those obtained from DFT calculations on a set of test data. A common approach is to extract structures from the same molecular dynamics simulations used to generate the training data, however, this might lead to artificially low errors [1]. This is especially relevant for our ML-FF as it should predict combinations of cations and Al distributions not included in the training data. The new structures used as test data is listed in Table 2.

Figure 2(a), shows the standard way of reporting the correlation, however, this procedure might hide the errors, especially if the energy span in which the test data is high. Instead, we plot the ML error, defined as the difference between the ML-FF predicted energy and the DFT energy, see Figure 2(b). Similar plots for the forces can be found in Figure 2(c-d). The ML-FF demonstrates low errors with an

RMSE of 0.94 meV/atom and 0.092 eV/Å for the energies and forces respectively.

Supplementary Table 2: Overview of systems included in the test data for Figure 2.

| Supercell | Si/Al | Cu/Al | $[Cu(NH_3)_2]^+$ | $NH_4{}^+$ |
|---|---|---|---|---|
| 2×1×1 | 6.2 | 0.4 | 4 | 6 |
| 2×1×1 | 4.1 | 0.29 | 4 | 10 |
| 2×1×1 | 7 | 0.56 | 5 | 4 |
| 3×1×1 | 107 | 1 | 1 | 0 |
| 3×1×1 | 26 | 0.75 | 3 | 1 |
| 3×1×1 | 8 | 0.33 | 4 | 8 |
| 3×1×1 | 7.3 | 0.61 | 8 | 5 |

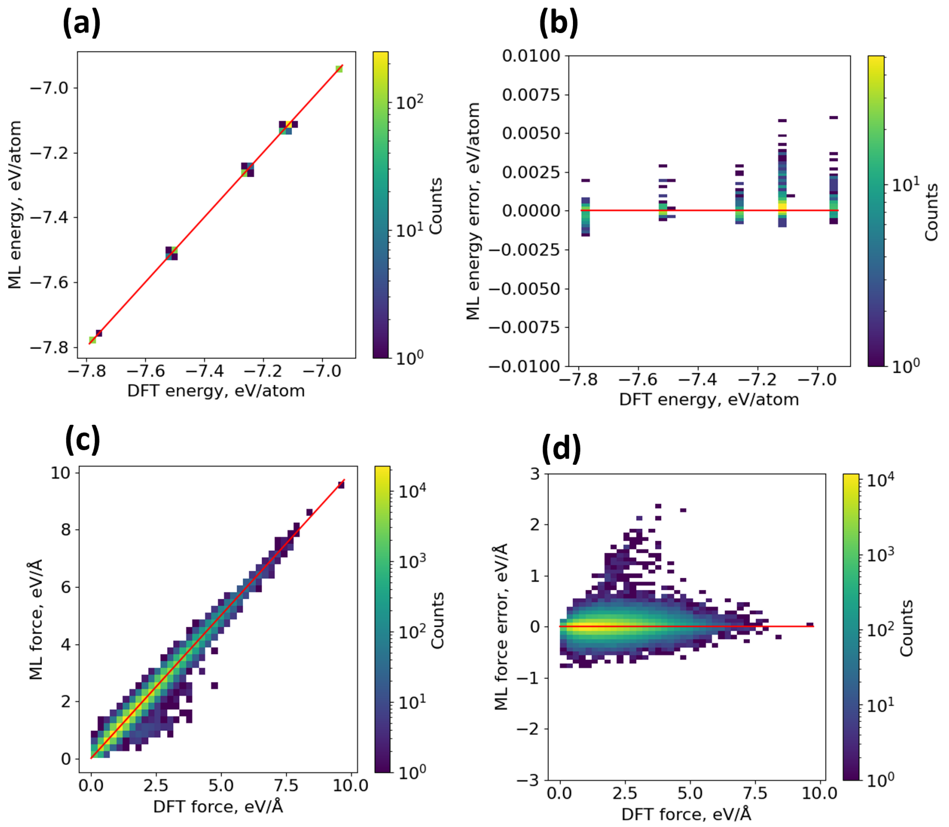


Supplementary Fig. 2: Correlation between predicted (a-b) and between predicted ML-FF forces and DFT forces (c-d). ML-FF error is the ML-FF predicted value subtracted from the DFT calculated value. n=700 and n=23850 for energies and forces respectively. Source data are provided as a Source Data file.

Given the long-range interactions in the system, it is important to test whether the ML-FF can extrapolate to larger supercells. For production runs, the ML-FF is used to simulate up to 3×3×3 supercells, which consist of thousands of atoms, where DFT calculations are not feasible. Instead, we validate on increasingly larger supercells, with the results shown in Figure 3 and the test data in Table 3. Note that the ML-FF has not been trained on the largest supercells (2×2×1 & 4×1×1) included in the test. The fact that the ML-FF accurately predicts energies and forces for these larger systems

demonstrates its applicability to larger supercells.

Supplementary Table 3: Overview of systems included in the test data for Figure 3.

| Supercell | Si/Al | Cu/Al | $[Cu(NH_3)_2]^+$ | $NH_4{}^+$ |
|---|---|---|---|---|
| 1×1×1 | 11 | 0.33 | 1 | 2 |
| 1×1×1 | 11 | 0.67 | 2 | 1 |
| 1×1×1 | 4.14 | 0.29 | 2 | 5 |
| 2×1×1 | 6.2 | 0.4 | 4 | 6 |
| 2×1×1 | 4.1 | 0.29 | 4 | 10 |
| 2×1×1 | 7 | 0.56 | 5 | 4 |
| 3×1×1 | 26 | 0.75 | 3 | 1 |
| 3×1×1 | 8 | 0.33 | 4 | 8 |
| 3×1×1 | 7.3 | 0.61 | 8 | 5 |
| 2×2×1 | 6.2 | 0.4 | 8 | 12 |
| 2×2×1 | 7.5 | 0.55 | 10 | 8 |
| 4×1×1 | 6.2 | 0.4 | 8 | 12 |
| 4×1×1 | 7.5 | 0.55 | 10 | 8 |

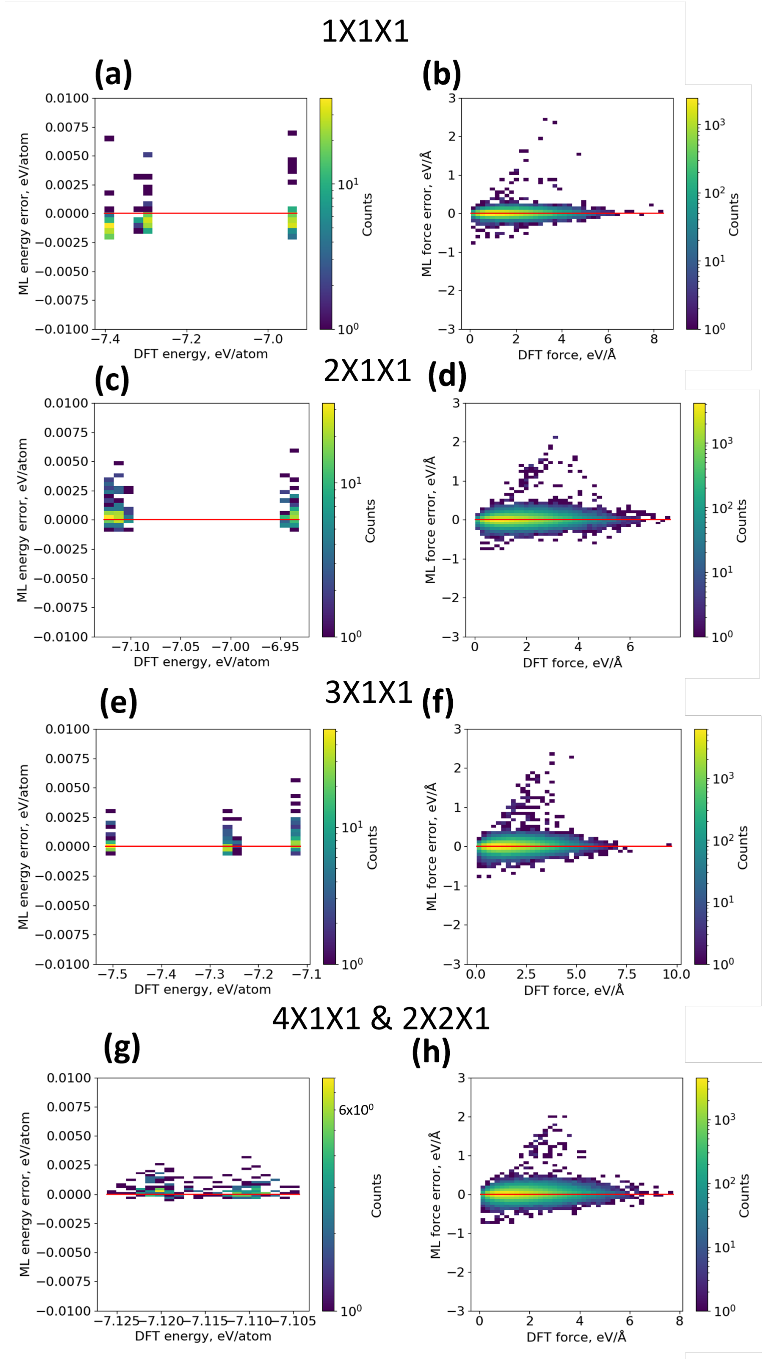


Supplementary Fig. 3: Correlation between predicted ML-FF energy and DFT energy and between predicted ML-FF forces and DFT forces for a supercell of 1×1×1 (a-b), 2×1×1 (c-d), 3×1×1 (e-f) and 4×1×1 2×2×1 (g-h). The root mean squared errors (RMSE) are (a) 1.35 meV, n=300 (b) 0.069 eV/Å, n=4900 (c) 1.13 meV/atom, n=300 (d) 0.091 eV/Å, n=86500 (e) 0.84 meV/atom, n=300 (f) 0.083 eV/Å, n=118700 (g) 0.76 meV/atom, n=200 and (h) 0.092 eV/Å, n=112600. ML-FF error is the ML-FF predicted value subtracted from the DFT calculated value. Source data are provided as a Source Data file.

## Collective variables

The Collective Variable (CV) used in the well-tempered metadynamics simulations is illustrated in Figure 4. The CV describes the diffusion of the $[Cu(NH_3)_2]^+$ complex through the eight-membered ring connecting the two cages. An axis going through the eight-membered ring is defined. From the Cu ion, a line is drawn intersecting the axis at a 90 degree angle. The CV value is determined by the position along the line at which it intersects, illustrated by a red dot. The middle of the eight-membered ring corresponds to a value of 0, and the CV value can be either positive or negative, depending on the location of the Cu ion.

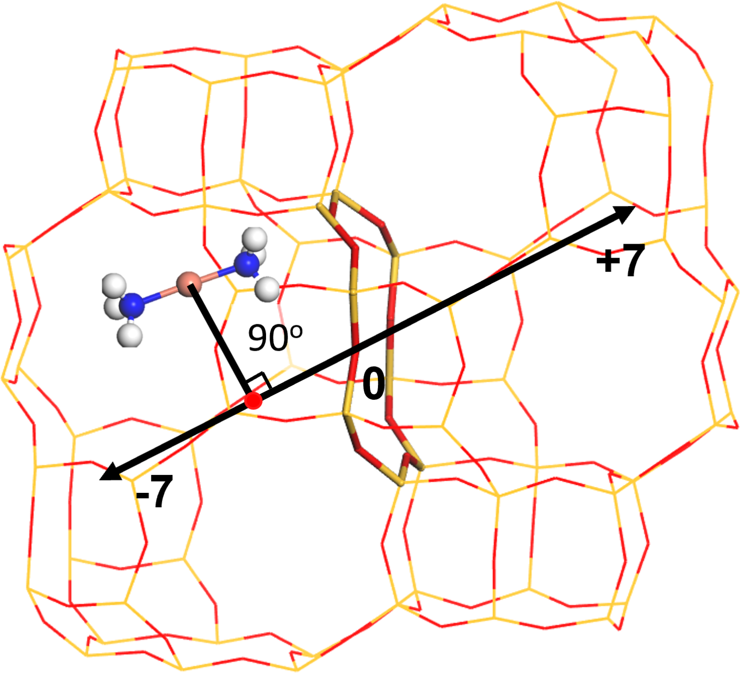


Supplementary Fig. 4: Illustration of the collective variable used in the well-tempered metadynamics simulations. Atomic color codes: H(white), N(blue), O(red), Si(yellow) and Cu(Bronze).

The CV's used for the two-dimensional well-temperated metadynamics simulations (Figure 4 in the main text) is illustrated in Figure 5. Figure 5(a) is the unit cell used in the simulations with selected cages highlighted. Figure 5(b-c) illustrates the CV for both $[Cu(NH_3)_2]^+$ and $NH_4{}^+$ and is the same as shown in Figure 4

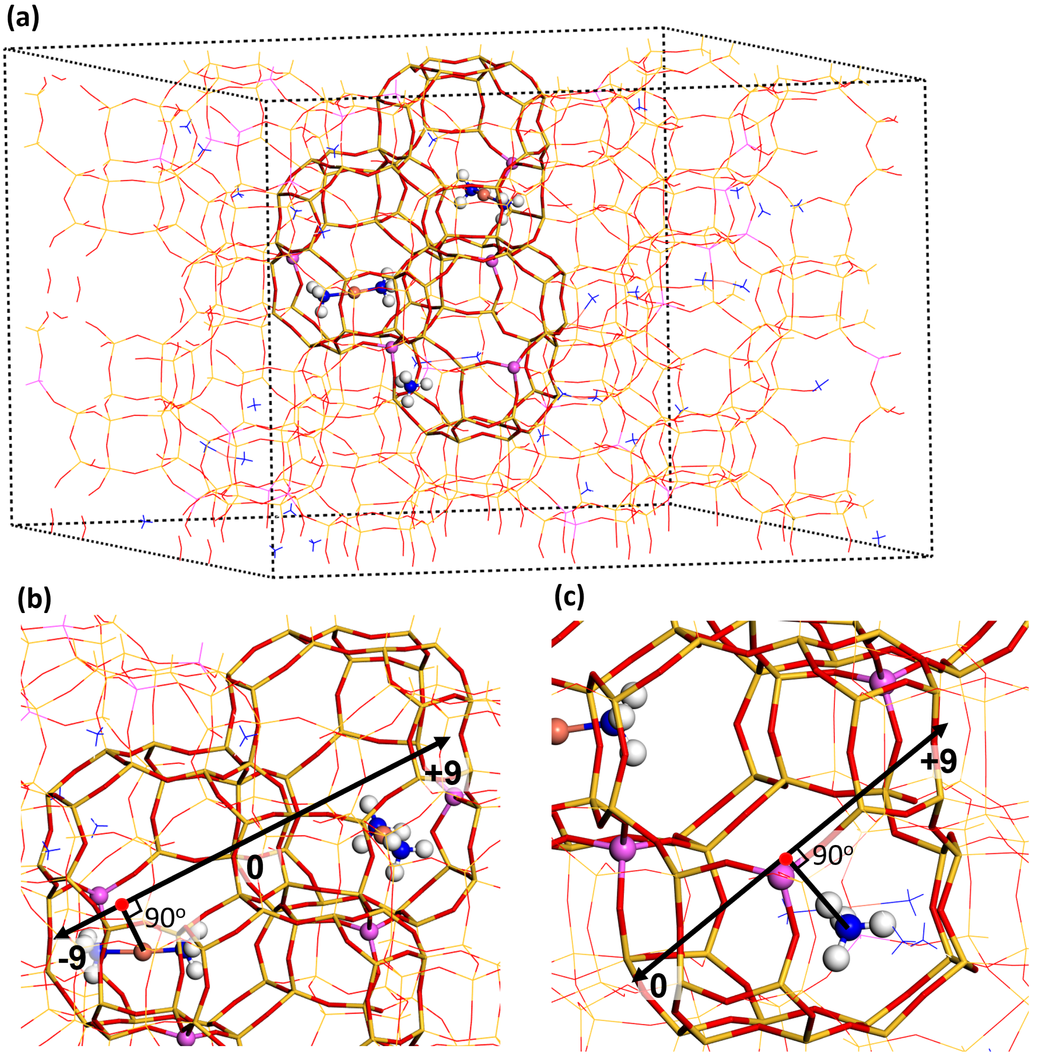


Supplementary Fig. 5: Illustration of the collective variables (CV) used in the two-dimensional well-tempered metadynamics simulations. (a) Overview of the structure used in the simulation. (b) CV for the $[Cu(NH_3)_2]^+$ complex. (c) CV for the $NH_4{}^+$ ion. Atomic color codes as in Supplementary Fig. 4 with Al(purple).

## Distribution of Al and counter ions

The supercells are based on a hexagonal CHA cage consisting of 36 silicon atoms and 72 oxygen atoms. The Al and counter ions ($[Cu(NH_3)_2]^+$ and $NH_4{}^+$) are distributed as follows. Firstly, the Al ions are randomly distributed ensuring no Al-O-Al bonds (Löwenstein's rule[2]). Secondly, each $[Cu(NH_3)_2]^+$ and $NH_4{}^+$ ions are assigned to an Al ion and placed in the corresponding cage, thus making certain that all

cages are initially charge-neutral.

The average fraction of paired $[Cu(NH_3)_2]^+$ complexes indicated with a dashed line in Figure 4 of the main text are computed as follows. Firstly, a 6×6×6 supercell is constructed, after which the Al ions are randomly distributed. Secondly, the $[Cu(NH_3)_2]^+$ ions are randomly assigned to an Al ion and are placed in the corresponding CHA cage. Thirdly, the fraction of paired $[Cu(NH_3)_2]^+$ is computed. The maximum number of $[Cu(NH_3)_2]^+$ complexes in one cage is set to 2. This procedure is repeated 100 times and the mean value is reported.

# Supplementary Discussion

## Inclusion of long-ranged interactions

Previous AIMD simulations have observed that the free energy increases significantly as the $[Cu(NH_3)_2]^+$ ion diffuses into an adjacent cage that does not share the same Al ion[3, 4]. This highlights the importance of the Coulomb interactions between the anionic Al-site and the cationic $[Cu(NH_3)_2]^+$ complex. Here we do additional calculations to explore the decay of the energy as $[Cu(NH_3)_2]^+$ move away from the Al-site.

A 10×1×1 supercell of a rhombohedral unit cell is constructed where the $[Cu(NH_3)_2]^+$ ion is put at different locations. Note that due to the increased size of the unit cell, single-point calculations are carried out. The $[Cu(NH_3)_2]^+$ is put in the middle of the cage, for each calculation. Thus, the only parameter that is changing is the distance between $[Cu(NH_3)_2]^+$ and the Al-site. Figure 6 shows the energy as a function of distance and the energy increases monotonically up to 45 Å. Note that the calculations employ periodic conditions, thus the reason why the energy flattens is attributed to interaction with the Al-site in the periodic image. This test stresses the importance of including long-range interactions in an ML-FF model for the simulation of Cu-CHA.

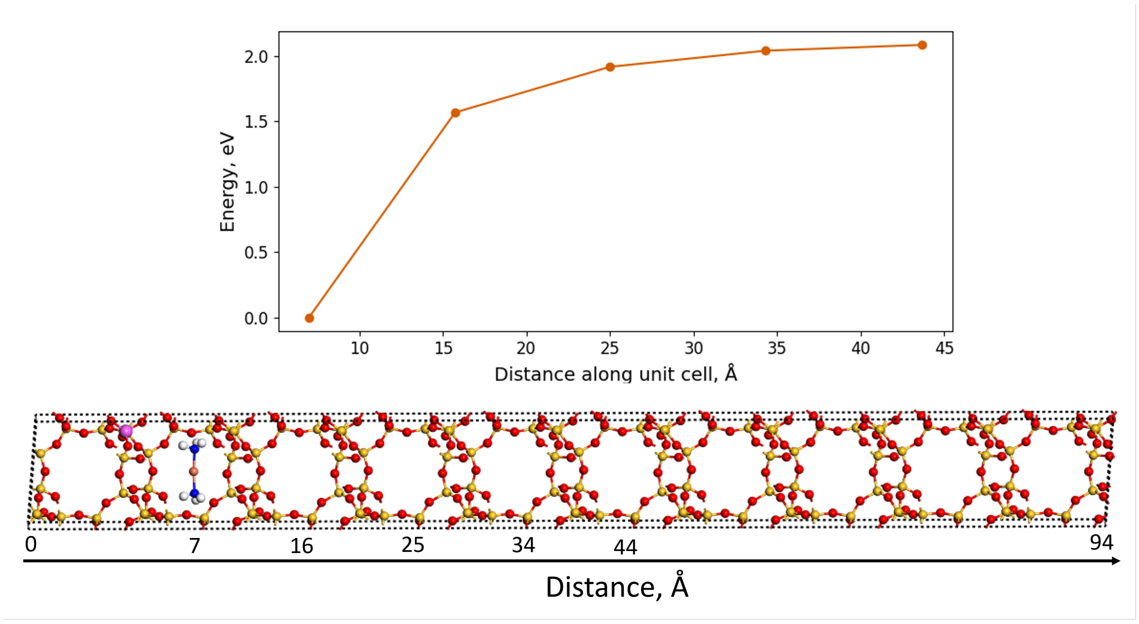


Supplementary Fig. 6: Energy for a $[Cu(NH_3)_2]^+$ complex placed at different distances from the the Al-site. Atomic color codes as in Supplementary Fig. 5. Source data are provided as a Source Data file.

## Importance of explicit long-ranged interactions

The deep potential long-range model (DPLR) from DeepMD-kit used in this study extends to their standard deep potential (DP) model, by augmenting long-range electrostatic interactions. Thus the energy predicted in the DPLR model can be expressed as a sum[5]:

$$E = E_{sr} + E_{G_t} \tag{1}$$

$E_{sr}$ is the short-range contribution from the standard DP model and $E_{G_t}$ is the long-ranged electrostatic energy. To examine the significance of the long-ranged electrostatic interactions, a DP model was trained using the same training data, and parameters as in the DPLR model, but without explicit long-ranged interactions.

Free energy landscapes were calculated for the case of *VI: Si/Al 13**(Figure 2(a) in the main text), with the result shown in Figure 7(a). For this example, the eight-membered ring connecting the two cages does not have an Al ion Hence, the $[Cu(NH_3)_2]^+$ complex is far from the Al-ion in the paired state. The standard DP model predicts the paired state to be 0.26 eV, more stable when compared to the DPLR model. This suggests that the DP model does not capture the destabilizing that arises when the distances between $[Cu(NH_3)_2]^+$ and the Al ion increases.

To isolate the effect of the $[Cu(NH_3)_2]^+$ diffusion away from an Al ion, a simplified system with a single

Al ion was constructed. The free energy landscape is reported in Figure 7(b) and the structure including the CV in Figure 7(c). As the distance between the Al ion and the $[Cu(NH_3)_2]^+$ complex increases, the DPLR model again predicts a higher relative free energy. However, the difference is less pronounced than in Figure 7(a). This may be attributed to the higher density of Al, $NH_4{}^+$, and $[Cu(NH_3)_2]^+$ ions in 7(a), which introduces additional interactions that can be more challenging to capture accurately. The errors in the free energy landscapes were estimated by analyzing the evolution of the free energy differences along the trajectories. The errors were found to be within about 0.1 eV.

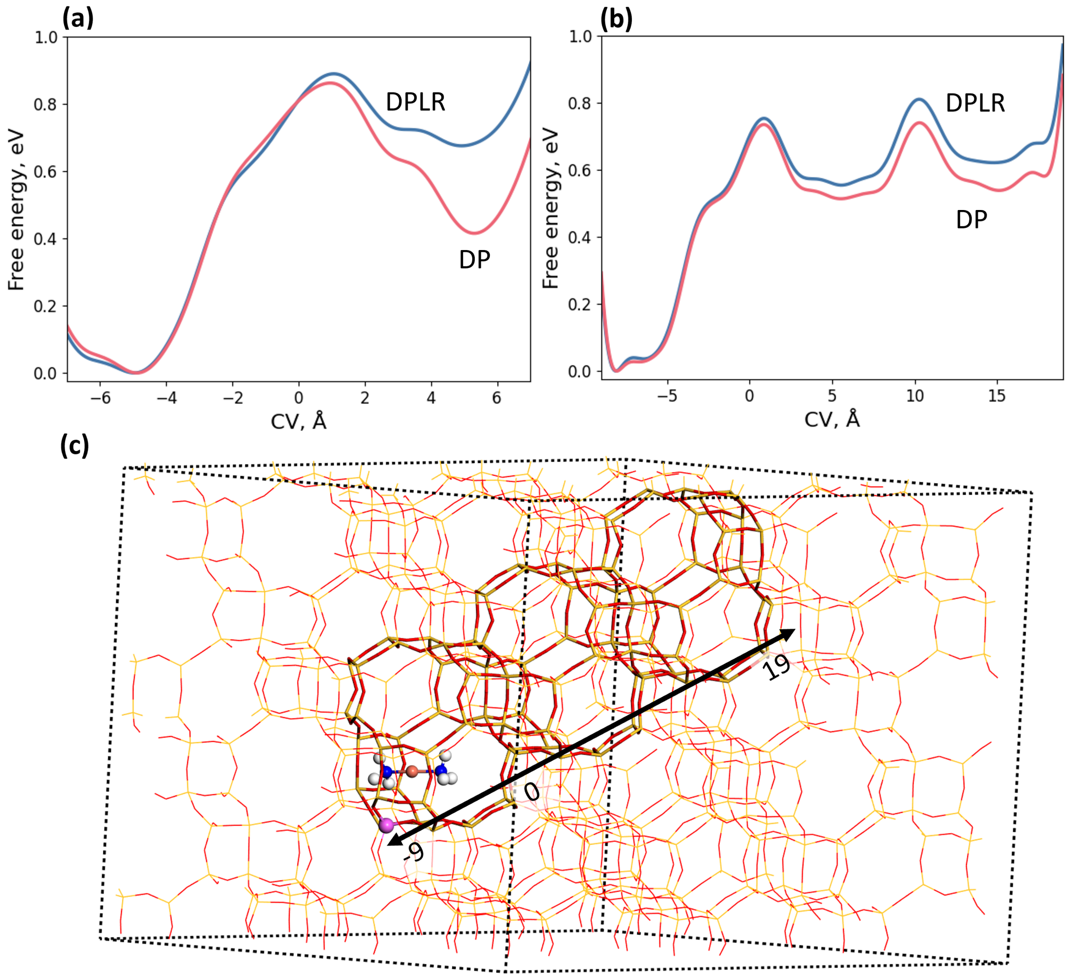


Supplementary Fig. 7: Free energy landscapes calculated using metadynamics for (a) for the pairing of two $[Cu(NH_3)_2]^+$ complexes and (b) for a $[Cu(NH_3)_2]^+$ complex diffusion away from an Al ion with structure and CV shown in (c). Atomic color codes as in Supplementary Fig. 5. Source data are provided as a Source Data file.

MD simulations were performed with the DP model for a system with a Si/Al ratio of 5 and Cu/Al ratio of 0.5, with the mean squared displacement (MSD) results shown in Figure 8. The simulation for the DPLR model is the same as shown in Figure 6 in the manuscript. The DP model predicts an increased diffusivity compared to the DPLR model for both the $[Cu(NH_3)_2]^+$ complexes and the $NH_4{}^+$ ions. As

shown in Figure 7, the DPLR model predicts higher free energies, which restricts the diffusion of the $[Cu(NH_3)_2]^+$ complexes (and likely also the $NH_4^+$ ions), resulting in the observed higher diffusivity for the DP model.

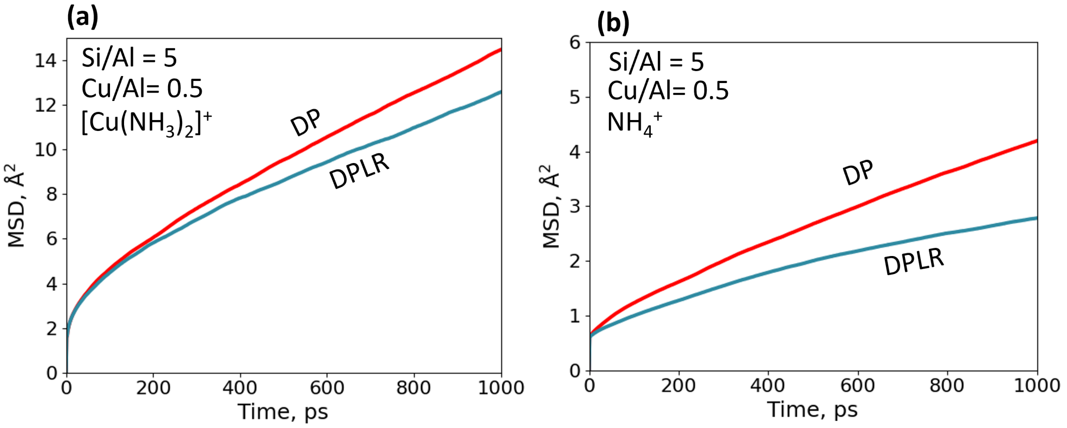


Supplementary Fig. 8: Mean square displacement for (a) $[Cu(NH_3)_2]^+$ and (b) $NH_4^+$ for Si/Al = 5 and Cu/Al 0.5 comparing the long-ranged model with an ML-FF model without long-range interaction included. Source data are provided as a Source Data file.

## Pair correlation functions

Pair correlation functions (PCF) are computed for Cu-Cu, Al-Cu, Al-N, N-N, and Cu-N where N is the nitrogen in $NH_4^+$. The PCF provide average information on the relative location of the species during the simulation. The results for Si/Al = 5 and Cu/Al = 0.5 are shown in Figure 9(a), 9(b) and 9(c). The computed PCF is an average over a 5 ns simulation.

The PCF for Al-N shows a large peak at 3.9 $\AA$ (Figure 9(a)). During the simulation, $NH_4^+$ preferably stays inside the eight-membered ring as illustrated in Figure 9d. Thus, the first peak in Figure 9(a) is the distance between an Al ion in the eight-membered and the $NH_4^+$ ion in the same ring. The narrow shape of the peak indicates that $NH_4^+$ does not move as much as $[Cu(NH_3)_2]^+$ for which the peaks are broad. For Al-Cu, the first peak has a maximum at 4.5 Å, thus the Cu complex is located further away from the Al ion as compared to $NH_4^+$. The broadness of the peak shows that $[Cu(NH_3)_2]^+$ is mobile within the cage.

Interestingly, the first peak for the Al-Cu PCF has a fine structure consisting of three smaller peaks. A zoom of this peak is shown for a simulation with the same number of $[Cu(NH_3)_2]^+$ ions, but varying the Si/Al ratio in Figure 9(b). Hence any difference observed is due to the increase or decrease in the number of Al and $NH_4^+$ ions. As the number of Al ions is changing, the difference in intensity between the PCFs should not be compared but instead the intensity difference between the three small peaks. In

the fine structure, the first peak at 3.5 Å is larger for a high Si/Al ratio, while it decreases as the Si/Al ratio is lowered. Hence as Si/Al ratio is lowered, the potential energy surface becomes flatter and the $[Cu(NH_3)_2]^+$ stay on average further away from the Al ion. Each Al ion shares three cages, thus the fine structure originates from these three different locations of $[Cu(NH_3)_2]^+$ relative to the Al ion.

The PCF plots for Cu-Cu, N-N and Cu-N are reported in Figure 9(c). For Cu-Cu, the peak at 5.5 Å corresponds to paired $[Cu(NH_3)_2]^+$ complexes and the peak at 7.9 Å corresponds to two $[Cu(NH_3)_2]^+$ complexes in nearby cages. The increase in the intensity of the Cu-Cu peak from the first to the second peak, suggests that the $[Cu(NH_3)_2]^+$ ions repel each other and that the paired state is unstable with respect to the separated case. The N-N PCF shows a large peak at 6.2 Å, hence the $NH_4{}^+$ ions can stay closer than the Cu complexes. The distance of 6.2 Å corresponds to the $NH_4{}^+$ ions being in two eight-membered rings next to each other. Interestingly the Cu-N profiles do not show any clear patterns, which probably is related to the fact that they occupy two different places, namely in the cage and inside the eight-membered ring.

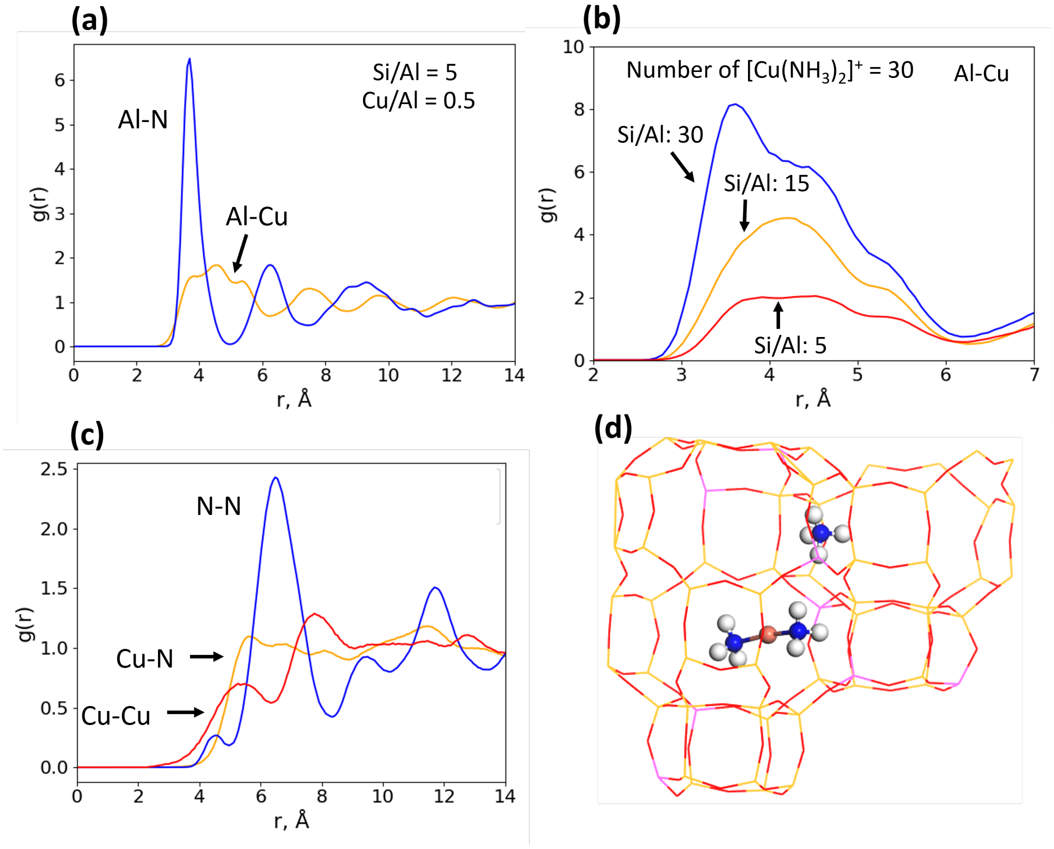


Supplementary Fig. 9: Pair correlation function (PCF) of (a) Al-Cu, Al-N with Si/Al = 5 and Cu/Al = 0.5. (b) Cu-Cu with Si/Al = 5, and Cu/Al = 0.25, 0.5 and 0.75. (c) Cu-Cu, N-N and Cu-N with with Si/Al = 5 and Cu/Al = 0.5. (d) Structure showing the typical location of the counter ions. Atomic color codes as in Supplementary Fig. 5. Source data are provided as a Source Data file.

## Paired Cu complexes - effect of Al and $NH_4^+$

To investigate the impact of Al and $NH_4^+$ ions on the fraction of paired Cu, unbiased simulations are performed with the same number of $[Cu(NH_3)_2]^+$ ions but with different Si/Al ratios. The Cu/Al ratio is adjusted to ensure the same number of $[Cu(NH_3)_2]^+$ complexes with the given Si/Al ratio, which is set to 30 complexes. The results are shown in Figure 10. The computed fraction of paired Cu is an average of 2 (SiAl = 5) or 3 (Si/Al = 15 & 30) simulations. A ratio of 5, results in a higher simulated fraction of paired Cu ions compared to Si/Al ratios of 15 and 30. This observation suggest that a higher abundance of $NH_4^+$ and Al ions enhances the stability of paired Cu complexes, in line with our metadynamics simulations. The difference between a Si/Al ratio of 15 and 30 is negligible, however, this may be explained by the smaller difference between the number of Al ions in the two cases. For a Si/Al ratio of 5, there are 162 Al ions, while it is 60 and 31 for an Si/Al ratio of 15 and 30, respectively. Note that the computed average fraction of paired $[Cu(NH_3)_2]^+$ complexes (dashed line) across the different Si/Al ratios is similar and can not capture the large difference in the simulated fraction of paired $[Cu(NH_3)_2]^+$ complexes. This further proves the importance of Al and $NH_4^+$ ions for the stability of paired configurations.

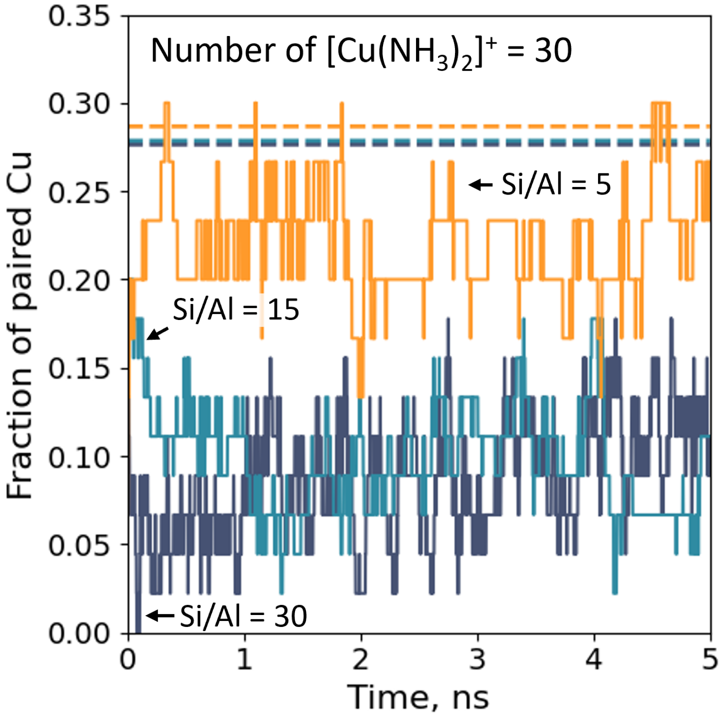


Supplementary Fig. 10: Fraction of paired $[Cu(NH_3)_2]^+$ ions as a function of simulation time for simulation with a fixed number of $[Cu(NH_3)_2]^+$ complexes but different Si/Al ratios. The dashed line represents the fraction of paired $[Cu(NH_3)_2]^+$ if they were distributed randomly. Source data are provided as a Source Data file.

## Mean square displacements for single species

The mean square displacement (MSD), is typically computed as an average over all $[Cu(NH_3)_2]^+$ complexes. In Figure 11(a-c), the MSD is computed for each $[Cu(NH_3)_2]^+$ complex and plotted as a dashed line. The solid line is the average of all $[Cu(NH_3)_2]^+$ complexes and is the result presented in Figure 6 in the main text. The MSD for the single $[Cu(NH_3)_2]^+$ complexes range from $\sim$ 1-2 Å$^2$ to 60 Å$^2$, thus, some complexes stay in their original cage, whereas others diffuse long distances. The results highlight the heterogeneous nature of Cu-CHA.

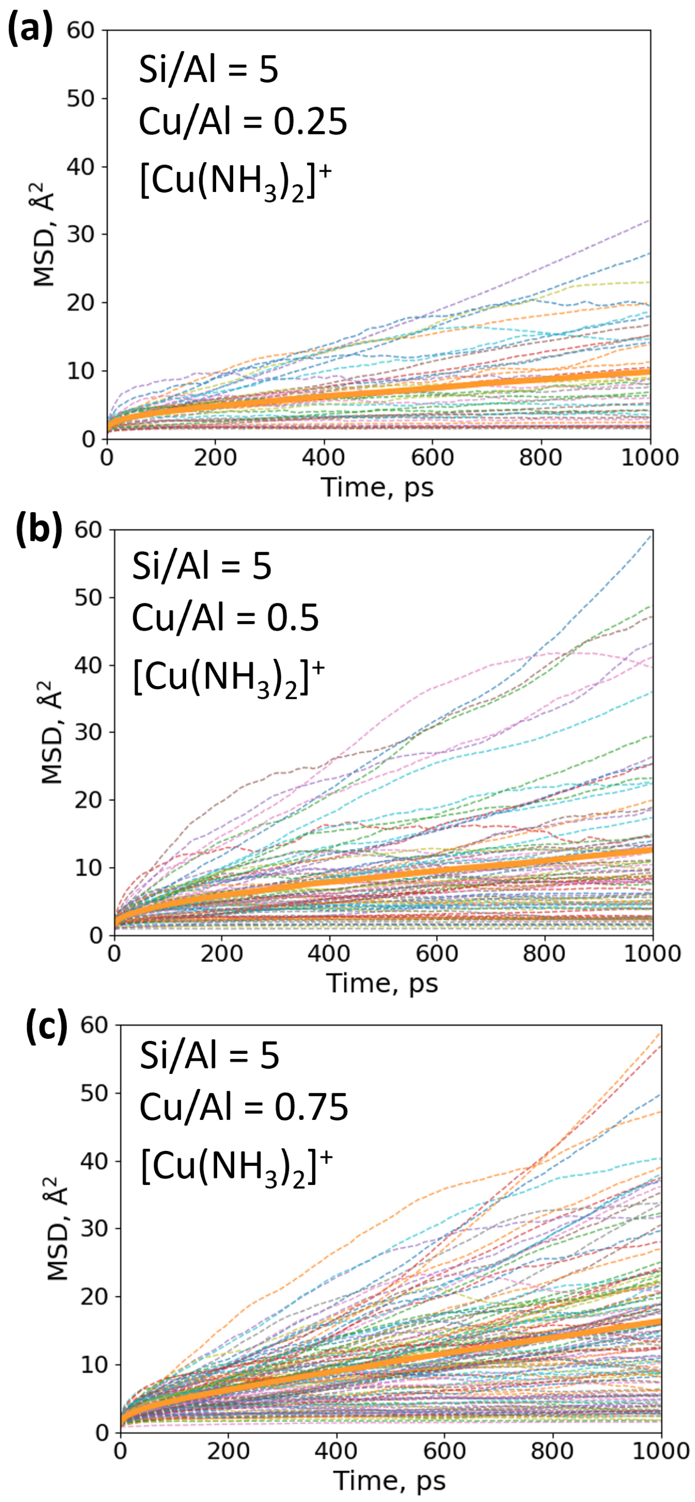


Supplementary Fig. 11: Mean square displacement for all $[Cu(NH_3)_2]^+$ complexes (dashed line) and the averaged one (solid line). Si/Al = 5 for a Cu/Al of (a) 0.25, (b) 0.5 and (c) 0.75. Source data are provided as a Source Data file.

# Supplementary References


1. Maxson, T., Soyemi, A., Chen, B. W. J. & Szilvási, T. Enhancing the Quality and Reliability of Machine Learning Interatomic Potentials through Better Reporting Practices. *J. Phys. Chem. C* **128,** 6524–6537 (2024).

2. Löwenstein, W. The Distribution of Aluminum in the Tetrahedra of Silicates and Aluminates. *Am. Min.* **39,** 92–96 (1954).

3. Paolucci, C. *et al.* Dynamic multinuclear sites formed by mobilized copper ions in NOx selective catalytic reduction. *Science* **357,** 898–903 (2017).

4. Millan, R., Cnudde, P., van Speybroeck, V. & Boronat, M. Mobility and Reactivity of $Cu^+$ Species in Cu-CHA Catalysts under $NH_3$-SCR-$NO_x$ Reaction Conditions: Insights from AIMD Simulations. *JACS Au* **1,** 1778–1787 (2021).

5. Zhang, L. *et al.* A deep potential model with long-range electrostatic interactions. *J. Chem. Phys.* **156,** 124107 (2022).
